# Relationship Between Lifestyle and Physical Fitness Among Older Women with Sarcopenia

**DOI:** 10.3390/ijms26052205

**Published:** 2025-02-28

**Authors:** Jun-Young Sung, Moon Jin Lee, Jiyoun Kim

**Affiliations:** 1 Department of Exercise Rehabilitation, Gachon University, 191 Hambakmoe-ro, Yeonsu-gu, Incheon 13120, Republic of Korea; sjy7067@gmail.com (J.-Y.S.); mjlee01@korea.ac.kr (M.J.L.); 2 Department of Physical Education, College of Education, Korea University, 145 Anam-ro, Swongbuk-gu, Seoul 02841, Republic of Korea

**Keywords:** female, sarcopenia, sociodemographic factors, lifestyle

## Abstract

This cross-sectional study aimed to identify the interactions between lifestyle-related, diagnostic, and physical strength-related sarcopenia factors. The study included 512 female participants aged 60–100 years from Incheon, Republic of Korea, recruited from 12 institutions. Participants engaged in the study from June to August 2023. We administered questionnaires on demographic characteristics and health indicators and undertook physical measurements, including grip strength and body composition. Hierarchical regression analysis and two-way analysis of variance were conducted to examine the association between sarcopenia and the examined variables. Statistical significance was set at *p* < 0.05. Hierarchical regression analysis of the variables affecting sarcopenia showed each characteristic’s effect: Model 1 (basic characteristic): R^2^, 0.391; *p* < 0.001; Model 2 (Model 1 + additional characteristics): R^2^, 0.427; *p* < 0.001. Hierarchical regression analysis of diagnostic and fitness factors affecting sarcopenia also showed an effect on sarcopenia (Model 1 (basic characteristics): R^2^, 0.318; *p* < 0.001; Model 2 (Model 1 + body composition): R^2^, 0.419; *p* < 0.001; Model 3 (Model 2 + fitness factors): R^2^, 0.664; *p* < 0.001). This study enhances the understanding of sarcopenia by investigating its connections with sociodemographic factors, lifestyle choices, and physical activity. The study underscores that lifestyle factors sustainably influence sarcopenia while confirming its correlation with fitness-related factors. Notably, this study highlights the results that muscle function is very important in preventing sarcopenia and that continuous physical activity and types of physical activity affect it.

## 1. Introduction

Globally, the population aged ≥ 65 years is steadily rising and is forecasted to reach approximately 1.5 billion by 2050, constituting approximately 16% of the total population [1]. Aging brings about numerous changes within the human body, one of the most prominent being a decline in muscle mass. Standard muscle mass in healthy adults makes up approximately 42% of the total body mass; however, this decreases to approximately 27% during the aging process [2].

Decreased muscle mass is accompanied by a functional decline in muscle strength [3] and causes morphological changes in body composition, such as changes in skeletal muscle, fat, and bony tissues [4]. This loss increases the risk of fractures, physical disabilities, chronic diseases, hematologic malignancy, and death [5]. Sarcopenia is a musculoskeletal disease accompanied by a gradual decrease in muscle mass and muscle strength. In 2010, the concept of sarcopenia was introduced, and in 2016, it was recognized as an independent disease by the International Classification of Diseases, Tenth Revision, Clinical Modification (ICD-10-CM) codes [6].

Sarcopenia can also affect the prognosis of other chronic diseases, such as cancer, diabetes mellitus, and cardiovascular disease [7]. Similar to these chronic diseases, sarcopenia reduction is also closely related to activity and diet. With age, food intake decreases significantly, which runs in parallel with a decrease in resting energy consumption [8]. Malnutrition among older adults means that most do not meet their energy or nutritional requirements, which leads to sarcopenia. However, the malnutrition spectrum also includes overnutrition, which leads to metabolic disorders and obesity [9]. Sarcopenia is diagnosed based on low muscle mass (LMM) and low muscle strength (LMS) [10]. The European Working Group on Sarcopenia in Older People recommends using the strength, ambulation, rising from a chair, stair climbing, and history of falling (SARC-F) questionnaire (five-measure instrument: strength, walking assistance, standing up from a chair, climbing stairs, and falling) [11] or the Ishii measuring instrument (including age, grip strength, and calf circumference) [12].

The prevalence of sarcopenia in women aged ≥65 years in community healthcare settings has been reported to be as high as 29%, and falls occur in 11–50% of adults >80 years of age [13]. In a study of women aged ≥65 years in Korea, the prevalence of sarcopenia was 22.1% [14]. Korea is one of the fastest-aging countries worldwide, and the demand for medical care and welfare for individuals aged ≥65 years is constantly increasing. However, sufficient information on the factors influencing the development of sarcopenia and their interactions has not been provided, and research on its association with physical activity is particularly lacking. Physical activity plays a crucial role in maintaining muscle mass and strength, yet it tends to decline naturally with aging. Therefore, a more detailed analysis is needed to examine the impact of physical activity levels on the onset of sarcopenia. Furthermore, studies considering the complex interactions among various lifestyle factors, sarcopenia diagnostic criteria, and physical performance remain insufficient.

In Korea, new research related to sarcopenia is necessary. An evaluation of domestic research trends showed that most previous studies—such as those by Jang [15] and Park [16]—only analyzed the prevalence and status of sarcopenia with the frequency and interactive effect of each variable. Therefore, this study aimed to identify the complex interactions among the participants’ lifestyle-related factors, diagnostic factors for sarcopenia, and physical strength factors to identify the factors that have a major influence on sarcopenia to provide guidance for the prevention of sarcopenia in individuals aged ≥65 years.

## 2. Results

Table 1 shows the demographic and clinical characteristics of the participants (total, 512; normal, 197; possible sarcopenia, 148; sarcopenia, 69; severe sarcopenia, 98). One-way analysis of variance (ANOVA) of the characteristics revealed statistically significant differences in all variables according to sarcopenia status (age, height, weight, body fat mass, body mass index (BMI), appendicular skeletal muscle (ASM), *p* < 0.001; disease, *p* = 0.012).

Table 2 shows the results of the two-way ANOVA of the variables influencing sarcopenia and fitness. All the variables influencing sarcopenia showed statistically significant differences; in particular, the ASM (age, *p* = 0.012; sarcopenia, *p* < 0.001; age × sarcopenia, *p* = 0.017), the short physical performance battery (SPPB) (age, *p* < 0.001; sarcopenia, *p* < 0.001; age × sarcopenia, *p* = 0.003), the hand grip (HG) (sarcopenia, *p* < 0.001), and the timed up and go (TUG) test showed statistically significant differences in the analysis of sarcopenia by age group (age, *p* < 0.001; sarcopenia, *p* < 0.001; age × sarcopenia, *p* = 0.016). In addition, all the factors related to fitness showed statistically significant differences. In particular, both dorsalflexion (DF) (age, *p* = 0.018; sarcopenia, *p* < 0.001; age × sarcopenia, *p* = 0.005) and the 2-min walk test (age, *p* < 0.001; sarcopenia, *p* = 0.002; age × sarcopenia, *p* = 0.044) showed statistically significant differences in the analysis of sarcopenia by age group.

Table 3 presents the results of the hierarchical regression analysis of the characteristic factors affecting sarcopenia. In Model 1 (basic characteristics), statistically significant results were found for age (*B*, 0.054; *t*, 6.271; *p* < 0.001), residential area (*B*, −0.042; *t*, −2.214; *p* = 0.028), and physical activity type (*B*, 0.072; *t*, 2.500; *p* = 0.013). In Model 2 (Model 1 + additional characteristics), statistically significant results were found for age (*B*, 0.045; *t*, 4.973; *p* < 0.001), residential area (*B*, −0.044; *t*, −2.338; *p* = 0.020), type of physical activity (B*B* 0.063; *t*, 2.159; *p* = 0.032), income level (*B*, −0.110; *t*, −2.517; *p* = 0.013), and alcohol consumption (*B*, 0.095; *t*, 2.724; *p* = 0.007). The Durbin–Watson value of the model in Table 3 was 1.956, which was close to 2, and all values were below variance inflation factor (VIF) 5.

Table 4 shows the results of the hierarchical regression analysis of the sarcopenia diagnostic and fitness factors. The analysis of Model 1 (basic characteristics) revealed statistically significant results for age (*B*, 0.066; *t*, 11.374; *p* < 0.001), regular physical activity (*B*, 0.191; *t*, 2.047; *p* = 0.041), and hyperlipidemia (*B*, −0.366; *t*, −3.830; *p* < 0.001). In contrast, that of Model 2 (Model 1 + body composition) revealed statistically significant results for age (*B*, 0.045; *t*, 6.846; *p* < 0.001), diabetes mellitus (*B*, 0.237; *t*, 2.413; *p* = 0.016), hyperlipidemia (*B*, −0.267; *t*, −2.943; *p* = 0.003), ASM (*B*, −0.364; *t*, −3.913; *p* < 0.001), calf circumference (CC) (*B*, −0.047; *t*, −2.055; *p* = 0.041), and percentage body fat (*B*, 0.020; *t*, 2.240; *p* = 0.026). The analysis of Model 3 (Model 2 + fitness factors) revealed statistically significant results for the ASM (*B*, −0.312; *t*, −4.243; *p* < 0.001), HG (*B*, −0.057; *t*, −6.924; *p* < 0.001), PF (*B*, 0.026; *t*, 2.256; *p* = 0.025), single leg stance test (*B*, −0.007; *t*, 2256; *p* = 0.007), 2 min walk test (*B*, −0.004; *t*, −2.516; *p* = 0.012), CC (*B*, −0.184; *t*, −8.444; *p* < 0.001), and SPPB (*B*, 0.095; *t*, 2.724; *p* = 0.007). The Durbin–Watson value of the model in Table 3 was 1.747, which was close to 2, and all values were below VIF 5.

## 3. Discussion

To provide guidelines for the prevention of sarcopenia in 65-year-olds ≥, this study aimed to identify complex interactions among participants’ lifestyle-related factors, sarcopenia diagnostic factors, and physical strength factors. As a result of the study, the interaction of each variable related to sarcopenia was confirmed, particularly, the complex interactions between lifestyle-related factors and physical strength factors were confirmed. The findings can help develop targeted treatment strategies for patients with sarcopenia.

Aging results in a significant reduction in muscle mass, which adversely affects the quality of life and autonomy in older adults [17]. Recent research indicates that skeletal muscle functions as an endocrine organ, releasing various physiological substances known as myokines, which are essential for physical activity [18]. One notable myokine, Secreted Protein Acidic and Rich in Cysteine (SPARC), has been identified as playing a critical role in curbing fat accumulation while fostering muscle regeneration and hypertrophy during the aging process [19]. SPARC’s activation is mediated through the phosphatidylinositol 4,5-bisphosphate 3-kinase (PI3K) signaling pathway linked to Insulin-like Growth Factor 1 (IGF-1) [20], making it vital for the prevention of sarcopenia. Additionally, other myokines, such as interleukin 6 (IL-6) and brain-derived neurotrophic factor (BDNF), have been implicated in the modulation of sarcopenia [21].

This study did not analyze the biological mechanisms found in patients with sarcopenia. However, its novelty lies in analyzing the factors that were most influential in sarcopenia through systematic analysis. Additionally, it has the advantage of presenting new results to sarcopenia researchers using statistical analyses that are not used in existing sarcopenia studies. According to the results of this study, differences in sociodemographic factors, regular physical activity, and types of physical activity were associated with sarcopenia. In particular, it was confirmed that the area of residence, physical activity, and income level played a major role in sarcopenia in terms of lifestyle-related factors. Sarcopenia has become a major social problem globally, having left a significant impact on healthcare and social security systems around the world [3]. The main causes of sarcopenia include aging, sociodemographic factors, and lifestyle factors [22].

Gao et al. [23] reported that high educational levels especially influence the prevention of sarcopenia, arguing that governmental educational support is needed for individuals aged ≥65 years with low educational levels. However, in this study, no association was found between the educational levels of the participants and their families and the prevalence of sarcopenia, which is considered to be the result of the establishment of a universal public education system and a lifelong learning system in Korea (senior university, government facility education). Other sociodemographic factors associated with sarcopenia include living in residential areas, comorbidities, income levels, and alcohol consumption. These findings correspond with those of a previous study [24] that found that social activities and income levels of individuals aged ≥65 years vary based on their residential areas and housing types and that the lower their physical activity levels, the higher their frequency of alcohol consumption.

Previous studies have indicated that physical activity in individuals aged ≥65 years is essential for preventing chronic diseases and sarcopenia [13]. Physical activity is also associated with the presence or absence of social activity and also affects the time spent at home, determining the income of individuals aged ≥65 years. These results may affect participants’ mental health, leading to depression, ultimately leading to overall physical weakness, and may lead to a vicious cycle, including further worsening of physical fitness [25]. Previous research has demonstrated that physical activity and exercise enhance the secretion of myokines, which are crucial for maintaining a healthy body. Furthermore, older adults who engage in higher levels of physical activity exhibit elevated concentrations of myokine factors in their bloodstream [17]. The findings of this study, analyzed through two-way ANOVA, corroborate the differences in physical fitness variables as influenced by age and the level of sarcopenia among participants. These results serve as a basis for determining the association between physical activity and myokine factors without blood factors analysis. In a study conducted on older women by Da Rocha et al., a decline in physical function and activity led to an increase in BMI, weight, and the risk of sarcopenia [26]. The results of the hierarchical regression analysis also confirmed the relationship between body composition, fitness factors, and sarcopenia. In particular, the hierarchical regression analysis of the ASM showed significant differences between the fitness factors in Model 2, (including body composition factors) and Model 3, (including fitness factors), confirming the relationship between sarcopenia and fitness factors.

However, excessive body fat composition can cause muscular obesity, and if an individual is obese, this complicates the diagnosis of sarcopenia [27]. Therefore, certain factors limit the diagnosis of sarcopenia, and follow-up studies using skin autofluorescence and various measurement tools for accurate diagnosis should be conducted in the future. Finally, as suggested by international clinical practice guidelines, diet and physical activity have been shown to be essential for the management of sarcopenia [22]. Regarding nutrition, individuals aged ≥65 years need direct monitoring and dietary surveys to ensure proper intake of protein and vitamin D, as well as energy balance [28].

This study had some limitations, such as the absence of a dietary survey and its cross-sectional design. It was also not possible to investigate the presence or absence of hypertension among the participants, so there is a limit to the investigation regarding the relationship between comorbidities and sarcopenia. Moreover, the inclusion of a dietary survey would have clarified the association between obesity and sarcopenia. Therefore, further investigations are warranted to explore the correlation between diet, lifestyle, and sarcopenia, whereas a longitudinal study, such as a follow-up cohort, would provide more comprehensive insights. Additionally, the fact that the study has no hematologic molecular analysis strongly highlights the need for follow-up studies. Therefore, it is deemed necessary to solidify the study’s results by analyzing hematologic factors related to sarcopenia, such as myokines, as follow-up studies.

## 4. Materials and Methods

### 4.1. Participants

This study included individuals aged 60–100 years residing in Incheon (downtown and outlying areas), Republic of Korea. The participants were 512 women who utilized 12 establishments catering to older adults, such as welfare and protection centers, and who agreed to participate in the study voluntarily. Participants with physical injuries, mobility difficulties, serious illnesses, or those who received surgical treatment in the last six months were excluded. Before study inclusion, all participants received a comprehensive explanation of the study, outlining the objectives, methodologies, and potential risks. They were explicitly informed of their right to withdraw from the study at any point without any negative consequences. Each participant provided written informed consent. The study protocol was approved by the GaChon University Institutional Bioethics Committee (approval no. 1044396-202301-HR-020-01) and adhered strictly to the principles outlined in the Declaration of Helsinki.

### 4.2. Measurement of Health and Physical Fitness Factors

The study was conducted between June and August 2023. Each participant completed a questionnaire regarding their demographic characteristics and physical fitness.

**Figure 1:**
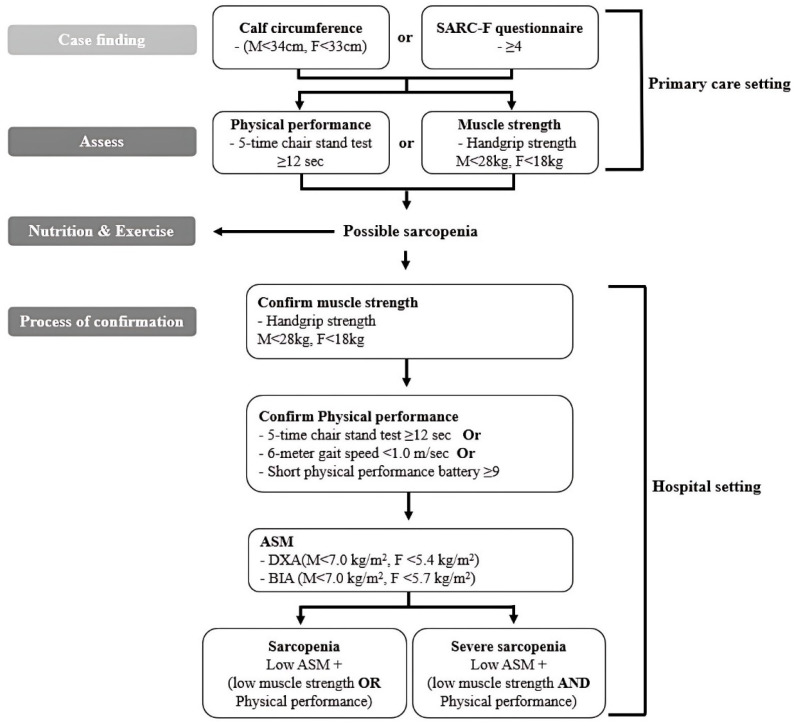
Asian diagnostic criteria for sarcopenia.

Fitness assessments were conducted in the same locations immediately following the administration of the questionnaire. They included an analysis of body composition (height, weight, body fat mass, ASM, and BMI). All measurements, except height, were performed using an InBody 720 (Biospace Cop, Seoul, Republic of Korea). The ASM was calculated after measuring the body composition with the following formula: skeletal muscle (kg) = 0.244 × body weight + 7.80 × height − 0.098 × age + 6.6 × sex + race − 3.3 [29]. We also measured blood pressure and CC. The diagnostic criteria for sarcopenia are shown in Figure 1 [30]. Physical fitness tests included the following:

1. HG: To measure HG, we utilized a hand dynamometer. We instructed the participant to exert maximal force on the dynamometer for approximately three seconds, ensuring that the arm was positioned at a right angle with the elbow close to the body.
2. PF: To measure PF, we used an isokinetic dynamometer. We instructed the participant to perform maximal plantar flexion against the resistance provided by the device, ensuring that the knee was extended, and the foot was positioned correctly on the footplate.
3. DF: To measure DF, we used an isokinetic dynamometer. We instructed the participant to perform maximal dorsiflexion against the resistance provided by the device, ensuring that the knee was extended, and the foot was properly aligned on the footplate.
4. SPPB: The SPPB was used to assess lower extremity function using three components: balance tests, a gait speed test, and a chair stand test. Each component was scored from 0 to 4, with higher scores indicating better performance and the total score ranging from 0 to 12.
5. TUG: The TUG test was used to measure mobility and balance. The participant began seated in a chair, stood up, walked a distance of three meters, turned around, walked back to the chair, and sat down. The time taken to complete the task was recorded, with shorter times indicating better functional mobility.
6. 2-min walking test: The 2-min walking test was used to measure the distance the participant could walk in two minutes. Without moving forward, their knees should rise to a specific marking height to recognize the walking cadence.

### 4.3. Statistical Analyses

All findings are reported as means ± standard deviations. Statistical analyses were conducted using SPSS version 26.0 (IBM Corp., Armonk, NY, USA). Group differences were evaluated using a one-way analysis of variance, followed by Tukey’s post-hoc test. Factors influencing sarcopenia and fitness were analyzed using a two-way ANOVA, and post-hoc analysis was performed using Tukey’s test. A hierarchical regression analysis was used to examine the associations between sarcopenia and the variables. Subsequently, a model was developed, with sarcopenia as the dependent variable and the rest as independent variables. Hierarchical regression analysis is a technique to evaluate the change in explanatory power at each step by adding independent variables incidentally, and the Durbin–Watson value and VIF are used to evaluate the appropriateness of the regression model. The Durbin–Watson test is an index to evaluate the autocorrelation between residuals in regression analysis, and the closer the value is to 2, the less autocorrelation there is. VIF measures the degree of multicollinearity, and a value of 10 or more is generally considered serious. Statistical significance was set at *p* < 0.05.

## 5. Conclusions

This study enriches the understanding of sarcopenia by exploring its associations with sociodemographic factors, lifestyle choices, and physical activity. As indicated by our study findings, lifestyle choices can have a lasting effect on sarcopenia, along with fitness factors. In particular, the importance of muscle function, or physical function, has been highlighted in preventing sarcopenia, and physical activity for this is also considered important. The insights gained from this study have the potential to enhance individuals’ quality of life, mitigate the risk of sarcopenia, and anticipate related comorbidities. Nonetheless, additional post-mortem investigations are warranted to validate these findings across diverse populations and elucidate the interplay between sarcopenia, obesity, blood factors, and dietary habits in Korea. Such endeavors hold promise for preventing sarcopenia and fostering the development of a robust community healthcare infrastructure, thereby enhancing the well-being of older adults.

## Figures and Tables

**Table 1:** The demographic characteristics of the participants.

Variables		Total n (%)	Normal n (%)	Possible n (%)	Sarcopenia n (%)	Severe n (%)	F	*p*
Age (years)	60–69	101 (19.7)	77 (39.1)	19 (12.8)	3 (4.3)	2 (2)	76.058	<0.001 ***
	70–79	165 (32.2)	85 (43.1)	45 (30.4)	22 (31.8)	13 (13.3)		
	80–89	204 (39.9)	32 (16.2)	72 (48.6)	35 (50.8)	65 (66.3)		
	>90	42 (8.2)	3 (1.5)	12 (8.1)	9(13.1)	18 (18.4)		
Height (cm)	<149.9	191 (37.3)	36 (18.3)	68 (45.9)	31 (44.9)	56 (57.1)	38.027	<0.001 ***
	150–159.9	287 (56.1)	134 (68.0)	76 (51.4)	37 (53.6)	40 (40.8)		
	>160	34 (6.6)	27 (13.7)	4 (2.7)	1 (1.5)	2 (2.1)		
Weight (kg)	<44.9	50 (9.8)	8 (4.1)	0 (0)	10 (14.5)	32 (32.7)	109.464	<0.001 ***
	45–49.9	66 (12.9)	20 (10.2)	2 (1.4)	18 (26.1)	26 (26.5)		
	50–54.9	123 (24)	50 (25.4)	14 (9.5)	36 (52.2)	23 (23.5)		
	>55	273 (53.3)	119 (60.3)	132 (89.1)	5 (7.2)	17 (17.3)		
BFM (%)	<19.9	12 (2.3)	2 (1)	0 (0)	6 (8.7)	4 (4.1)	92.899	<0.001 ***
	20–29.9	88 (17.2)	39 (19.8)	5 (3.4)	15 (21.7)	29 (29.6)		
	30–39.9	271 (52.9)	122 (61.9)	60 (40.5)	40 (58)	49 (50)		
	>40	141 (27.6)	34 (17.3)	83 (56.1)	8 (11.6)	16 (16.3)		
BMI (kg/m^2^)	<18.5	33 (6.5)	16 (8.1)	0 (0)	6 (8.7)	11 (11.2)	93.037	<0.001 ***
	18.6–24.9	197 (38.5)	108 (54.9)	20 (13.5)	4 (5.8)	65 (66.3)		
	25–29.9	189 (36.9)	63 (31.9)	91 (61.5)	13 (18.8)	22 (22.5)		
	>30	93 (18.1)	10 (5.1)	37 (25)	46 (66.7)	0 (0)		
ASM	<5.9	262 (51.1)	93 (47.2)	37 (25)	65 (94.2)	97 (98.9)	137.385	<0.001 ***
	6–8.9	222 (48.9)	103 (52.2)	111 (75)	4 (5.8)	1 (1.1)		
	>9	1	1 (0.6)	0 (0)	0 (0)	0 (0)		
Disease	Yes	301 (58.8)	103(52.2)	89 (60.1)	40 (58)	69 (97.9)	3.701	0.012 *
	No	211 (31.2)	94 (47.8)	59 (39.9)	29 (42)	29 (2.1)		
Total		512	197	148	69	98		

Values are means ± SD * *p* < 0.05, *** *p* < 0.001, by Tukey-test; Possible, possible sarcopenia; Severe, severe sarcopenia, BMI, body mass index, BFM, body fat mass, and ASM, appendicular skeletal muscle.

**Table 2:** The two-way ANOVA results of lifestyle factors affecting sarcopenia.

	Variable		60 s	70 s	80 s	90 s		F	*p*
1	ASM	Normal	6.37 ± 0.08	5.99 ± 0.08	5.83 ± 0.12	7.19 ± 0.41	A:	3.687	**0.012 ^+^**
		Possible	7.04 ± 0.16	6.53 ± 0.10	6.41 ± 0.08	6.26 ± 0.20	S:	62.439	**<0.001 ^###^**
		Sarcopenia	5.16 ± 0.49	5.34 ± 0.15	4.93 ± 4.68	4.42 ± 0.29	A × S:	2.272	**0.017** *
		Severe	5.12 ± 0.49	5.15 ± 0.19	4.87 ± 0.09	4.55 ± 0.17			
	CC	Normal	34.66 ± 0.28	34.03 ± 0.27	32.93 ± 0.43	34.00 ± 1.41	A:	1.148	0.329
		Possible	35.52 ± 0.56	34.13 ± 0.37	34.43 ± 0.30	34.50 ± 0.70	S:	35.216	**<0.001 ^###^**
		Sarcopenia	31.50 ± 1.41	32.05 ± 0.52	30.91 ± 0.44	28.50 ± 1.01	A × S:	1.828	0.061
		Severe	28.50 ± 1.73	30.36 ± 0.62	30.27 ± 0.31	30.81 ± 0.58			
	SPPB	Normal	11.83 ± 0.21	11.53 ± 0.20	11.29 ± 0.36	11.00 ± 1.85	A:	7.927	**<0.001 ^+++^**
		Possible	9.78 ± 0.42	9.47 ± 0.28	7.31 ± 0.26	8.25 ± 0.92	S:	23.961	**<0.001 ^###^**
		Sarcopenia	10.67 ± 1.07	9.77 ± 0.39	8.67 ± 0.38	6.50 ± 1.31	A × S:	2.808	**0.003** **
		Severe	8.50 ± 1.31	6.62 ± 0.51	6.44 ± 0.28	5.50 ± 0.58			
	TUG	Normal	7.44 ± 0.55	7.52 ± 0.52	9.11 ± 0.92	12.13 ± 4.82	A:	6.012	**0.001 ^+++^**
		Possible	10.87 ± 1.11	10.13 ± 0.72	12.53 ± 0.68	16.62 ± 2.40	S:	8.400	**<0.001 ^###^**
		Sarcopenia	8.25 ± 2.78	9.46 ± 1.03	10.54 ± 0.98	14.80 ± 3.41	A × S:	2.288	**0.016** *
		Severe	9.90 ± 3.41	15.48 ± 1.37	15.95 ± 0.71	15.50 ± 1.54			
2	HG	Normal	24.81 ± 0.45	22.71 ± 0.43	20.72 ± 0.76	20.39 ± 3.94	A:	1.671	0.173
		Possible	19.63 ± 0.90	19.05 ± 0.93	16.69 ± 0.55	14.18 ± 1.97	S:	16.829	**<0.001 ^###^**
		Sarcopenia	17.93 ± 2.27	19.47 ± 0.84	18.70 ± 0.80	16.64 ± 2.79	A × S:	1.084	0.373
		Severe	10.85 ± 2.79	14.47 ± 1.09	14.53 ± 0.59	13.27 ± 1.25			
	PF	Normal	17.89 ± 0.44	15.82 ± 0.42	13.77 ± 0.75	15.10 ± 3.89	A:	4.092	**0.007 ^++^**
		Possible	16.18 ± 0.89	12.78 ± 0.58	10.68 ± 0.53	10.06 ± 1.94	S:	10.986	**<0.001 ^###^**
		Sarcopenia	16.70 ± 2.24	13.71 ± 0.83	12.35 ± 0.79	11.56 ± 2.75	A × S:	1.268	0.252
		Severe	7.60 ± 2.75	9.20 ± 1.08	10.05 ± 0.58	7.87 ± 1.23			
	DF	Normal	15.27 ± 0.38	13.64 ± 0.36	11.04 ± 0.64	14.20 ± 3.34	A:	3.408	**0.018 ^+^**
		Possible	13.27 ± 0.76	9.62 ± 0.49	7.89 ± 0.46	5.99 ± 1.67	S:	15.413	**<0.001 ^###^**
		Sarcopenia	12.00 ± 1.92	10.25 ± 0.71	9.37 ± 0.68	7.54 ± 2.36	A × S:	2.664	**0.005** **
		Severe	4.40 ± 2.36	6.31 ± 0.93	7.88 ± 0.50	5.66 ± 1.06			
	2 min	Normal	97.70 ± 2.85	98.25 ± 2.71	83.66 ± 4.81	46.00 ± 24.99	A:	9.132	**<0.001 ^###^**
		Possible	81.47 ± 5.73	68.73 ± 3.73	47.31 ± 3.47	30.00 ± 12.49	S:	4.904	**0.002 ^++^**
		Sarcopenia	79.00 ± 14.43	75.50 ± 5.33	68.63 ± 5.10	28.00 ± 17.67	A × S:	1.947	**0.044** *
		Severe	55.50 ± 17.67	51.84 ± 6.93	48.53 ± 3.73	29.60 ± 7.90			

Values are means ± SD ^+^ *p* < 0.05, ^++^ *p* < 0.01, ^+++^ *p* < 0.001 by ages; ^###^ *p* < 0.001 by sarcopenia; * *p* < 0.05, ** *p* < 0.01 by ages × sarcopenia; A, age; S, sarcopenia; A × S, ages × sarcopenia; Possible, possible sarcopenia; severe sarcopenia; 1, the factor of sarcopenia; 2, the factor of fitness test; ASM, appendicular skeletal muscle; CC, calf circumference; HG, hand grip; PF, plantar flexion; DF, dorsalflexion; SPPB, short physical performance battery; TUG, timed up and go; 2 min, 2 min walking test.

**Table 3:** The results of characteristics factors affecting sarcopenia.

	Independent Variable	Model 1				Model 2			
		B	β	t	*p*	B	β	t	*p*
A	(Constant)	−3.222		−3.951	<0.001 ***	−2.902		−2.739	0.007 **
	Age	0.054	0.420	6.271	<0.001 ***	0.045	0.346	4.973	<0.001 ***
	Residential area	−0.042	−0.142	−2.214	0.028 *	−0.044	−0.149	−2.338	0.020 *
	Religion	0.043	0.053	1.035	0.302	0.054	0.066	1.276	0.203
	Education level	−0.083	−0.082	−1.415	0.158	−0.030	−0.030	−0.480	0.631
	Regular physical activity	0.154	0.044	0.844	0.400	0.056	0.016	0.300	0.764
	Type of physical activity	0.072	0.138	2.500	0.013 *	0.063	0.120	2.159	0.032 *
	Family					0.013	0.014	0.275	0.783
	House					0.012	0.008	0.168	0.867
	Education level of family					0.006	0.006	0.114	0.910
	Income					−0.110	−0.156	−2.517	0.013 *
	Drinking frequency					0.095	0.143	2.724	0.007 **
	Smoking frequency					0.124	0.021	0.410	0.682
	F (*p*)	25.504 (<0.001 ***)				14.415 (<0.001 ***)			
	R2	0.391				0.427			
	adjR2	0.376				0.398			

Significant difference, * *p* < 0.05, ** *p* < 0.01, *** *p* < 0.001; tested by hierarchical regression analysis. A (dependent variable: sarcopenia level); Model 1, basic characteristics; Model 2, Model 1 + additional characteristics.

**Table 4:** Results of hierarchical regression analysis of sarcopenia diagnostic factors.

	Independent Variable	Model 1				Model 2				Model 3			
		B	β	t	*p*	B	β	t	*p*	B	β	t	*p*
A	(Constant)	−4.213		−9.53	<0.001 ***	0.384		0.498	0.619	6.877		8.865	<0.001 ***
	Age	0.066	0.495	11.374	<0.001 ***	0.045	0.337	6.846	<0.001 ***	−0.003	−0.019	−0.388	0.698
	RPA	0.191	0.088	2.047	0.041 *	0.169	0.079	1.951	0.052	0.042	0.020	0.623	0.534
	Diabetes	0.203	0.084	1.940	0.053	0.237	0.098	2.413	0.016 *	0.049	0.020	0.632	0.528
	Hyperlipidemia	−0.366	−0.166	−3.830	<0.001 ***	−0.267	−0.121	−2.943	0.003 **	−0.131	−0.060	−1.815	0.070
	ASM					−0.364	−0.32	−3.913	<0.001 ***	−0.312	−0.274	−4.243	<0.001 ***
	CC					−0.047	−0.127	−2.055	0.041 *	−0.004	−0.012	−0.236	0.814
	Percent body fat					0.020	0.136	2.240	0.026 *	−0.008	−0.056	−1.154	0.249
	HG									−0.057	−0.28	−6.924	<0.001 ***
	PF									0.026	0.116	2.256	0.025 *
	DF									−0.022	−0.093	−1.686	0.093
	SLT									−0.007	−0.118	−2.711	0.007 **
	CSR									−0.003	−0.030	−0.827	0.409
	2 min									−0.004	−0.108	−2.516	0.012 *
	Timed up and go									−0.024	−0.117	−1.862	0.063
	Gait speed									0.012	0.023	0.388	0.698
	SPPB									−0.184	−0.427	−8.444	<0.001 ***
	F (*p*)	44.115 (<0.001 ***)				38.706 (<0.001 ***)				45.291 (<0.001 ***)			
	R2	0.318				0.419				0.664			
	adjR2	0.311				0.409				0.650			

Significant difference, * *p* < 0.05, ** *p* < 0.01, *** *p* < 0.001; tested by hierarchical regression analysis. A (dependent variable: sarcopenia level), sarcopenia; Model 1, basic characteristics; Model 2, Model 1 + body composition; Model 3, Model 2 + fitness factors; RPA, regular physical activity; ASM, appendicular skeletal muscle; CC, calf circumference; HG, hand grip; PF, plantar flexion; DF, dorsalflexion; SLT, single leg stance test; SPPB, short physical performance battery, 2 min, 2 min walking test; CHR, chair sit and reach.

## Data Availability

The datasets used and/or analyzed during the current study are available from the corresponding author on reasonable request.

## Abbreviations

The following abbreviations are used in this manuscript:

LMM	Low muscle mass
LMS	Low muscle strength
ASM	Appendicular skeletal muscle
BMI	Body mass index
CC	Calf circumference
HG	Hand grip
PF	Plantar flexion
DF	Dorsalflexion
SPPB	Short physical performance battery
TUG	Timed up-and-go
ANOVA	Analysis of variance
SPARC	Secreted Protein Acidic and Rich in Cysteine
PI3K	phosphatidylinositol 4,5-bisphosphate 3-kinase
IGF-1	Insulin-like Growth Factor 1
IL-6	interleukin 6
BDNF	Brain-derived neurotrophic factor
TNF-α	Tumor necrosis factor-α
